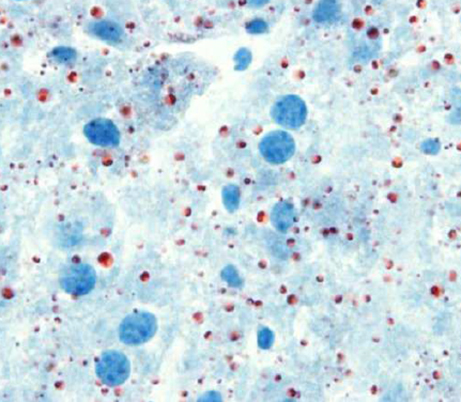# A role for bone mineralisation factors in obesity and diabetes

**Published:** 2014-12

Bone has emerged as an important endocrine regulator that can mediate the interplay between bone remodelling and energy metabolism. Ectonucleotide pyrophosphatase/phosphodiesterase-1 (NPP1) is a factor that regulates bone mineralisation and is elevated in individuals with insulin resistance, a condition associated with type 2 and obesity-related diabetes. Thus, this factor might play a role in the development of metabolic disease. Vicky MacRae and colleagues sought to investigate this by using *Enpp^−/−^* mice, which lack NPP1 and exhibit impaired bone metabolism. These mice showed a pronounced resistance to obesity and to the development of insulin resistance in response to chronic high-fat feeding. Moreover, *Enpp1^−/−^* mice exhibited increased levels of the bone-derived hormone osteocalcin, which increases β-cell proliferation and insulin secretion, thereby linking bone remodelling to metabolic homeostasis. These results support the involvement of NPP1 in the development of obesity and type 2 diabetes via its regulation of insulin sensitivity. The NPP1 pathway might hence represent a potential therapeutic target to treat insulin resistance in human metabolic disease. **Page 1341**

**Figure:**